# Correction: Cancer care in German centers of excellence during the first 2 years of the COVID-19 pandemic

**DOI:** 10.1007/s00432-024-06060-2

**Published:** 2025-01-18

**Authors:** Volker Arndt, Daniela Doege, Stefan Fröhling, Peter Albers, Hana Algül, Ralf Bargou, Carsten Bokemeyer, Martin Bornhäuser, Christian H. Brandts, Peter Brossart, Sara Yvonne Brucker, Tim H. Brümmendorf, Hartmut Döhner, Norbert Gattermann, Michael Hallek, Volker Heinemann, Ulrich Keilholz, Thomas Kindler, Cornelia von Levetzow, Florian Lordick, Ulf Peter Neumann, Christoph Peters, Dirk Schadendorf, Stephan Stilgenbauer, Thomas Zander, Daniel Zips, Delia Braun, Thomas Seufferlein, Gerd Nettekoven, Michael Baumann

**Affiliations:** 1 Division of Clinical Epidemiology and Aging Research (C070), Unit of Cancer Survivorship (C071), German Cancer Research Center (DKFZ), Im Neuenheimer Feld 280, 69120 Heidelberg, Germany; 2 Division of Translational Medical Oncology, German Cancer Research Center (DKFZ), National Center for Tumor Diseases (NCT), Heidelberg, Germany; 3 Department of Urology, Comprehensive Cancer Center, Center for Integrated Oncology (CIO Aachen, Heinrich Heine University Düsseldorf), Medical Faculty of the Heinrich Heine University Düsseldorf, Düsseldorf, Germany; 4 Comprehensive Cancer Center München, Institute for Tumor Metabolism, Klinikum rechts der Isar, Technical University of Munich,, Munich, Bavaria,, Germany; 5 Comprehensive Cancer Center Mainfranken, University Hospital Würzburg, Würzburg, Germany; 6 Medical Clinic and Polyclinic II, Center for Oncology, University Cancer Center Hamburg, University Medical Center Hamburg-Eppendorf, Hamburg, Germany; 7 Medical Clinic I, National Center for Tumor Diseases (NCT/UCC), University Hospital Carl Gustav Carus Dresden, TU Dresden, Dresden, Germany; 8 University Cancer Center (UCT) Frankfurt-Marburg, Frankfurt University Hospital, Frankfurt, Germany; 9 Medical Clinic III and Center for Integrated Oncology (CIO Aachen, Bonn, Cologne, Düsseldorf), University Hospital Bonn, Bonn, Germany; 10 Comprehensive Cancer Center Tübingen-Stuttgart, Department of Women’s Health, University Hospital Tübingen, Tübingen, Germany; 11 Medical Clinic IV and Center for Integrated Oncology (CIO Aachen, Bonn, Cologne, Düsseldorf), University Hospital of RWTH Aachen, Bonn, Aachen, Germany; 12 Comprehensive Cancer Center Ulm (CCCU), Department of Internal Medicine III, University Hospital Ulm, Ulm, Germany; 13 Department of Hematology, Oncology and Clinical Immunology, Comprehensive Cancer Center, Center for Integrated Oncology (CIO Aachen, Bonn, Cologne, Düsseldorf), Medical Faculty of the Heinrich Heine University Düsseldorf, Düsseldorf, Germany; 14 Clinic I for Internal Medicine, Center for Integrated Oncology (CIO Aachen, Bonn, Cologne, Düsseldorf), University Hospital Cologne, Düsseldorf, Germany; 15 Medical Clinic and Polyclinic III, LMU Hospital, Munich, Germany; 16 Charité Comprehensive Cancer Center (CCCC), Berlin, Germany; 17 University Cancer Center (UCT), University Medical Center Mainz, Mainz, Germany; 18 University Cancer Center Leipzig and Department of Medicine II, University of Leipzig Medical Center, Leipzig, Germany; 19 Department of Visceral and Transplantation Surgery, University Hospital of RWTH Aachen, Aachen, Germany; 20 Tumor Center Freiburg, Institute of Molecular Medicine and Cell Research, Freiburg, Germany; 21 West German Tumor Center (WTZ) Essen and Clinic for Dermatology, Essen University Hospital, Essen, Germany; 22 Comprehensive Cancer Center Tübingen-Stuttgart and Department of Radiation Oncology, University Hospital Tübingen, Tübingen, Germany; 23 German Cancer Research Center (DKFZ), Heidelberg, Germany; 24 German Cancer Society, Berlin, Germany; 25 Department of Internal Medicine I, University Hospital Ulm, Ulm, Germany; 26 German Cancer Aid, Bonn, Germany

Correction to: Journal of Cancer Research and Clinical Oncology (2022) 149:913–919

10.1007/s00432-022-04407-1

In this article the affiliation details for Author Hana Algül were incorrectly given as ‘Comprehensive Cancer Center TUM (CCCMTUM), Klinikum Rechts der Isar of the Technical University of Munich, Munich, Germany’ should have been ‘Comprehensive Cancer Center München, Institute for Tumor Metabolism, Klinikum rechts der Isar, Technical University of Munich, Munich, Bavaria, Germany’.

The content of the article remains unchanged.

The original article has been corrected.

